# A Lego-Like Reconfigurable Microfluidic Stabilizer System with Tunable Fluidic RC Constants and Stabilization Ratios

**DOI:** 10.3390/mi15070843

**Published:** 2024-06-28

**Authors:** Wuyang Zhuge, Weihao Li, Kaimin Wang, Zhuodan Chen, Chunhui Wu, Kyle Jiang, Jun Ding, Carl Anthony, Xing Cheng

**Affiliations:** 1 Guangdong-Hong Kong-Macau Joint Laboratory on Micro-Nano Manufacturing Technology, Department of Materials Science and Engineering, Southern University of Science and Technology, No. 1088 Xueyuan Blvd., Shenzhen 518055, China; wxz807@student.bham.ac.uk (W.Z.); li.weihao@u.nus.edu (W.L.); 12232063@mail.sustech.edu.cn (K.W.); 12132027@mail.sustech.edu.cn (Z.C.); wuch@sustech.edu.cn (C.W.); 2 Department of Mechanical Engineering, University of Birmingham, Edgbaston, Birmingham B15 2TT, UK; k.jiang@bham.ac.uk (K.J.); c.j.anthony@bham.ac.uk (C.A.); 3 Department of Materials Science and Engineering, National University of Singapore, 9 Engineering Drive 1, Singapore 117576, Singapore; msedingj@nus.edu.sg; 4 Yangtze Delta Region Institute of Tsinghua University, 705 Yatai Road, Jiaxing 314006, China

**Keywords:** modular microfluidic stabilizer system, 3D printing, fluidic circuit analogy, tunable stabilization ratio, droplet generation

## Abstract

In microfluidic systems, it is important to maintain flow stability to execute various functions, such as chemical reactions, cell transportation, and liquid injection. However, traditional flow sources, often bulky and prone to unpredictable fluctuations, limit the portability and broader application of these systems. Existing fluidic stabilizers, typically designed for specific flow sources, lack reconfigurability and adaptability in terms of the stabilization ratios. To address these limitations, a modular and standardized stabilizer system with tunable stabilization ratios is required. In this work, we present a Lego-like modular microfluidic stabilizer system, which is fabricated using 3D printing and offers multi-level stabilization combinations and customizable stabilization ratios through the control of fluidic RC constants, making it adaptable to various microfluidic systems. A simplified three-element circuit model is used to characterize the system by straightforwardly extracting the RC constant without intricate calculations of the fluidic resistance and capacitance. By utilizing a simplified three-element model, the stabilizer yields two well-fitted operational curves, demonstrating an R-square of 0.95, and provides an optimal stabilization ratio below 1%. To evaluate the system’s effectiveness, unstable input flow at different working frequencies is stabilized, and droplet generation experiments are conducted and discussed. The results show that the microfluidic stabilizer system significantly reduces flow fluctuations and enhances droplet uniformity. This system provides a new avenue for microfluidic stabilization with a tunable stabilization ratio, and its plug-and-play design can be effectively applied across diverse applications to finely tune fluid flow behaviors in microfluidic devices.

## 1. Introduction

Flow stabilization is a crucial component in fluid control strategies employed by microfluidic controllers. The flow stability has direct effects on the expected functional performance in a wide range of applications, such as droplet generation [1], concentration gradient generation [2], sample injection [3], cell transportation [4], and microfluidic sensors [5]. In order to maintain the flow stability in microfluidics, extensive research has been conducted on microfluidic stabilizers for several decades. For example, researchers have studied passive-type stabilizers, such as membrane-based [6,7,8] and air-based stabilizers [9,10,11], which could function independently of off-chip hardware and therefore facilitate portability. However, previous studies have predominantly focused on the design and evaluation of the functional elements. As the complexity of microfluidic systems evolves, there is a growing need for a modular paradigm [12,13] that supports modularized and standardized designs with more adequately fitted working curves and customized stabilization ratios. Therefore, modularized and standardized designs are desired for future microfluidic stabilizers.

Currently, researchers have made significant strides in developing passive-type microfluidic stabilizers, with innovative designs such as the hand-powered injector featuring a flexible PDMS membrane [3], which is particularly useful in resource-limited settings. Air-chamber-based microfluidic stabilizers were demonstrated as an effective solution for syringe pumps working at low input frequencies [10]. Despite these advancements, achieving a good stabilization ratio below 2% has not resolved the issues of modularization and customization. This has led to the creation of specialized, one-off designs of stabilizers with static stabilization curves, where altering the stabilization ratio entails a costly and time-consuming redesign of the mask for mold fabrication [14]. To solve those problems, modular design can provide compelling solutions. Modular design’s high reconfigurability allows for the use of standardized linkers and devices, facilitating the control of stabilization ratios and enabling the quick assembly of stabilizer systems with diverse working curves in a rapid and low-cost way [15,16,17].

A modular approach to microfluidics is predicated upon the principles of additive manufacturing, commonly referred to as three-dimensional printing (3DP) [18]. This advanced manufacturing technique facilitates the fabrication of intricate geometrical configurations [19] with reduced financial expenditure and diminished labor intensity relative to conventional production methodologies, such as injection molding or soft lithography [20]. These attributes render 3DP particularly amenable to the creation of microfluidic devices that are both modular and conform to standardized design principles—for example, 3D-printed microfluidic counterparts of the circuit elements (e.g., capacitor, diode, and transistor) [21] and Lego-like studs for standardized conformation of microfluidic devices [15,22]. Despite the reported progress of 3D printing in microfluidics, there is a notable absence of modular 3D-printed reconfigurable stabilizer systems. This gap signifies an opportunity for further research and development within the field.

In this work, we propose a novel microfluidic stabilizer system (MSS) featuring tunable stabilization ratios and RC constants fabricated by 3D printing and analyze its transient behaviors of stabilizing fluctuations for multiple input frequencies using circuit analysis methods. A pluggable, modularized Lego-like MSS with two levels of working combinations is presented. The modular design of the MSS allows for easy adjustments of flowrate amplitude modulations, and it can be easily connected to different pumps and microfluidic chips by its luer connector. This design enables the MSS to achieve flow stabilization with fluctuations of less than 1%. Moreover, a simplified circuit analysis model [23] is adopted to predict the stabilization ratio of the MSS, thereby offering a controllable working curve. Finally, we demonstrate that the MSS can stabilize the fluid flow in a cross-flow droplet generation device to significantly improve the uniformity of the droplets’ diameter.

## 2. Design and Fabrication

**Figure 1:**
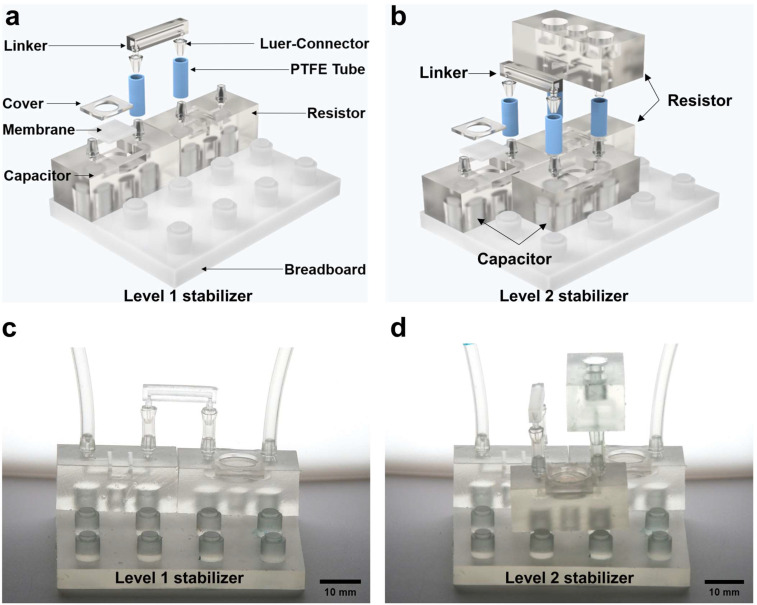
The design of the modular MSS: (**a**) illustration of level-1 Lego-like MSS; (**b**) illustration of level-2 Lego-like MSS; (**c**) photograph of level-1 Lego-like MSS with scale bar; (**d**) photograph of level-2 Lego-like MSS with scale bar.

We explored a series of designs (see Section S1 and Figure S1 in Supplementary Materials). The optimized design is shown in Figure 1a,b, which comprises a modular microfluidic capacitor, a modular microfluidic resistor, a modular linker and a fluid breadboard for flow connection (see Figure S2 in Supplementary Materials for more details). The assembled level-1 stabilizer and level-2 stabilizer are shown in Figure 1c,d.

In addition, the illustration also shows the capability for vertically cascaded connection. Specifically, the microfluidic stabilizer was assembled using three elements: (1) a main body printed at a 365 nm UV wavelength with a 3D printer (Asiga-Max, Ann Arbor, MI, USA), which is a 15×12×12 mm cube with 800 μm diameter micro-channels inside; (2) a 500 μm thick silicone rubber membrane (Ecoflex00-30, Smooth-On, USA), which is used to provide compliance to achieve capacitive behavior for fluid flows; and (3) a 11.8×11.8 mm 3D-printed upper cover with a height of 1.2 mm, which is used to fix the compliant ecoflex membrane. These three elements were assembled together using hydrophobic photoresist (RZ304, Rui Hong Electronic Chemicals, Suzhou, China or Leaftop 9311). Additionally, two luer connectors were designed at the top of the modular device to form connections to other devices via PTFE tubes. In order to assemble and organize the modular units, a 3×4-pillar breadboard is proposed. As shown in Figure 1, this design permits a rapid assembly of multiple microfluidic capacitors and microfluidic resistors by plugging through the luer connectors, allowing for the facile realization of various fluidic stabilizer combinations. By using the modular design, customized structures with different stabilization ratios can be assembled within 1 min. The system maintains its operational efficacy and stability even after multiple cycles of disassembly and reassembly, demonstrating its robustness and efficiency.

**Figure 2:**
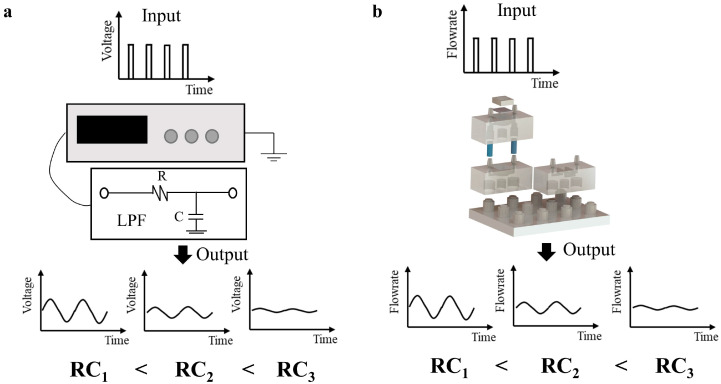
Schematic illustrations of outputs with different RC constants for (**a**) a classical electronic low-pass filter circuit and (**b**) a modular MSS.

The working mechanism of the proposed system with a flow source is illustrated in Figure 2b in analogy to an electrical low-pass filter in Figure 2a. Figure 2a shows the classical electrical low-pass filter consisting of a signal generator plus a simple RC circuit. When the input signal is filtered by the RC circuit, the output signal is described in the form of sine waves with a specific amplitude, which is determined by the RC constant of the filter circuit. In analogy, the proposed MSS could function similarly in a microfluidic circuit. As depicted in Figure 2b, for a given input flow through the pluggable MSS, the amplitude of the flowrate oscillation could be modulated accordingly with the RC constant of the MSS, with a larger RC constant resulting in a smaller flowrate oscillation. Furthermore, due to the reconfigurability of the modular design, a range of RC constants could be achieved by simply connecting a certain number of microfluidic capacitors and resistors to satisfy specific applications.

## 3. Experimental Setup and Fluidic Circuit Modelling

**Figure 3:**
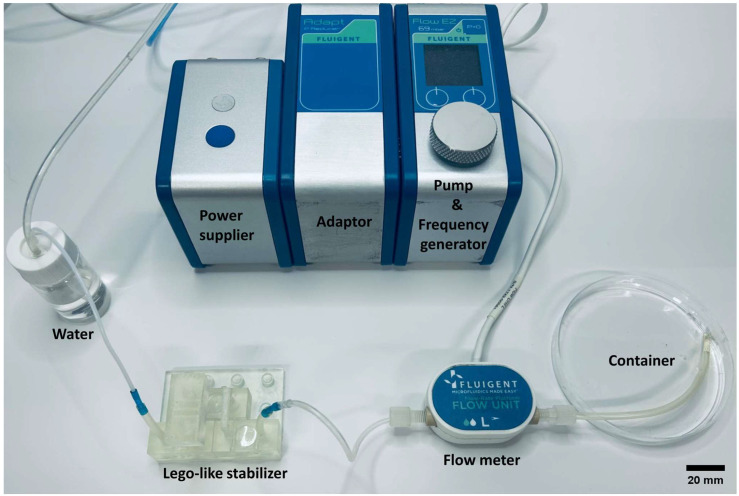
A photograph of the experimental setup for the characterization of the transient behavior of the fluidic system with an MSS.

To study how to control the stabilization ratios of the MSS using RC constants, an experiment is conducted. To compare the theoretical working curve of the MSS with the experimental stabilization ratios in the frequency domain, the system is studied at different working frequencies to extract the amplitude responses of the MSS. Figure 3 shows the experimental setup for the characterization of the transient behavior of a fluidic system with a MSS.

**Figure 4:**
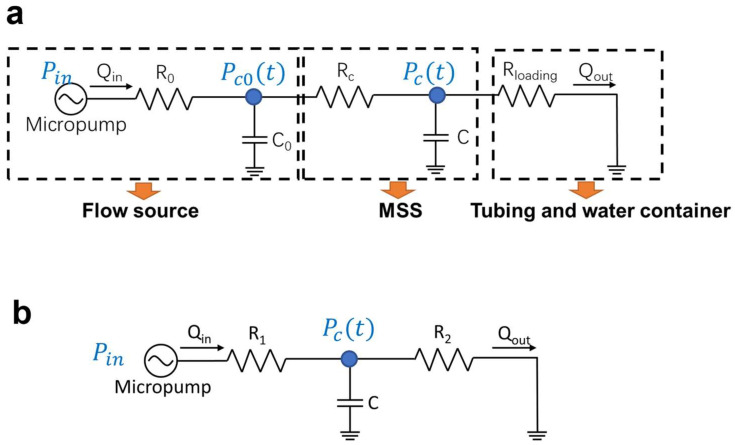
(**a**) An equivalent circuit diagram for the experimental setup; (**b**) a simplified three-element model.

The setup consists of several parts: a pump as the flow source (Fluigent, Kremlin-Bicêtre, France), an MSS, a flow sensor (Fluigent, Kremlin-Bicêtre, France), tubes, and a water container. The micropump is a gas pump with sine wave pressure output. The pulsing frequency of the micropump varies from 0.1 Hz to 5 Hz with the same pressure offset at 5 mbar and amplitude at 2 mbar. The water container provides a connection to the atmospheric pressure, which serves as the “ground” contact for the fluidic circuit. The equivalent fluidic circuit diagram for the fluidic setup in Figure 3 is shown in Figure 4a. To simplify the analysis for the MSS while maintaining the model accuracy, we further simplify the circuit diagram into a three-element model, shown in Figure 4b.

Using circuit analysis, the simplified model in Figure 4b has the following Kirchhoff current equation for the node (blue dot):

(1) $$\frac{P_{in}-P_{c}\left(t\right)}{R_{1}}=C_{overall}\frac{dP_{c}\left(t\right)}{dt}+\frac{P_{c}\left(t\right)}{R_{2}}$$

$$\mathrm{where}\;C_{overall}=\left(\frac{R_{c}}{R_{0}}C+C_{0}\frac{R_{c}}{R_{loading}}+C_{0}+C\right).$$

More details about the derivation of the equations are discussed in Section S2 of Supplementary Materials.

With the simplified circuit model, we then apply Thevenin’s theorem to the three-element model and convert it into a series RC circuit. In this study, we use features from a series RC circuit to predict the experimental outcomes. The transfer function of the RC circuit is then written in Equation (2) as:

(2) $$Stabilization\;ratio=H\left(j\omega \right)=\sqrt{\frac{1}{{\left(\omega RC\right)}^{2}+1}}=\frac{Amplitude\;of\;output\;signal}{Amplitude\;of\;input\;signal}$$

where *ω* means the angular frequency of the input signal, *R* refers to the fluidic resistance of the low-pass filter circuit, and *C* is the capacitance of the fluidic capacitor. The time constants *τ* of different levels of stabilizers are extracted (see Section S3 in Supplementary Materials). By using RC constants and Equation (2) to describe the transient behavior of the fluidic circuit, the relationship between the stabilization ratios and input frequencies is illustrated without extracting accurate values of the resistance or capacitance of the system.

## 4. Results and Discussion

### 4.1. Model Validation

**Figure 5:**
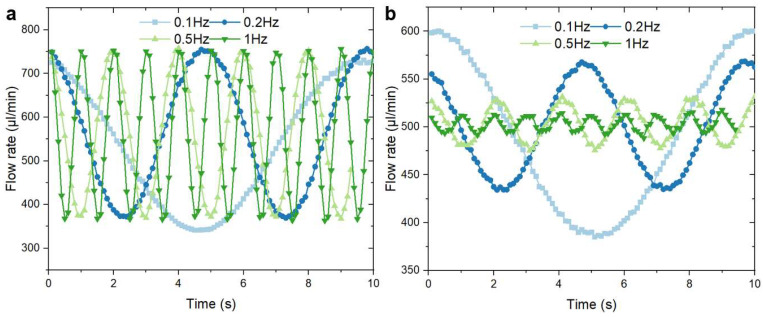
(**a**) The background output recorded from the flow sensor with 200 ± 5 µL/min amplitude when the input frequencies varying from 0.1 Hz to 1 Hz; (**b**) the amplitude response of the level-1 MSS at various driving frequencies.

As Equation (2) predicts that the stabilization ratios of the MSS are dependent on the flow frequency, we characterized the behaviors of the MSS in the frequency domain to verify the validity of the simplified model. Figure 5a shows the background output flowrates recorded from the flow sensor, which is driven by a sine pressure wave from the fluid controller without linking stabilizers. All the sine pressure waves have an amplitude of 2 mbar and an offset of 5 mbar in order to guarantee that the only variable is the input frequency. The average amplitude of the background signal is 200 ± 5 µL/min. The theoretical stabilization ratios at different working frequencies were calculated using Equation (2). The overall RC constant of the MSS was measured using the extraction method, which is detailed in Section S3 of Supplementary Materials. As shown in Figure 5a, the background flowrates oscillate with a fixed amplitude. Figure 5b depicts the experimentally obtained flowrate ratios of the level-1 stabilizer at various driving frequencies. It is obvious that the amplitude was reduced significantly when the system worked with the stabilizer. Meanwhile, the increase in working frequency leads to a reduction in amplitude and fluctuation.

**Figure 6:**
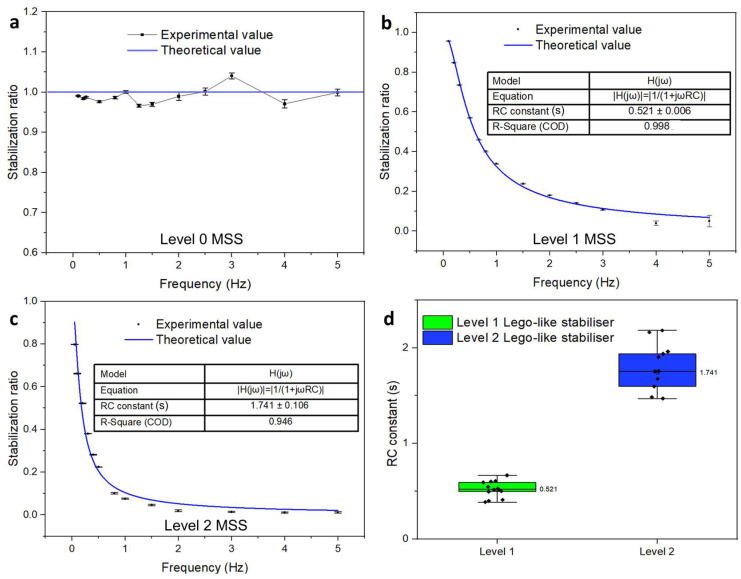
The stabilization ratios obtained from theoretical calculations (blue) and experimental measurements (black): (**a**) background amplitude response of the system as level-0 stabilizer; (**b**) working curve of level-1 Lego-like MSS; (**c**) working curve of level-2 Lego-like MSS; (**d**) the distribution of extracted RC constants.

The theoretical predictions of the stabilization ratios from the circuit analysis were calculated using Equation (2). The calculated ratios and the experimentally obtained ratios are listed and compared in Figure 6.

As shown in Figure 6, Equation (2) serves as a simplified model with decent accuracy. Figure 6a is the amplitude response of the background fluidic circuit. The RC constant of the level-0 stabilizer system equals 0 s; thus, the theoretical amplitude response is 1 at different frequencies. A comparison between the theoretical working curves and the experimental working curves of the level-1 stabilizer and level-2 stabilizer is illustrated in Figure 6c,d. According to Figure 6c,d, the fitted working curves have an R-square coefficient of over 0.946 when compared with the experimental value. Figure 6d illustrates the distribution of the extracted RC constants of the MSS (see Section S3 in Supplementary Materials for the extracting process). The extracted mean value of the RC constant of the level-1 stabilizer is 0.521 ± 0.006 s, which leads to a minimum stabilization ratio of 1.4%. Meanwhile, the RC constant of the level-2 stabilizer is 1.741 ± 0.106 s, which leads to a minimum stabilization ratio of less than 1%. However, there are still noticeable discrepancies when the working frequency is higher than 4 Hz, as shown in Figure 6b, which can be ascribed to several causes, such as imperfections in the data acquisition, device parameter extraction, and oversimplification of the circuit models. Despite the existence of these error sources, the model described in this work provides decent accuracy and is a convenient quantitative tool for the design of an MSS with controllable stabilization ratios. Comparisons between different stabilizer systems are listed in Table 1. The MSS can provide a less than 1% stabilization ratio with modular combinations, possessing well-predicted adjustable working curves while simultaneously maintaining a relatively optimal stabilization ratio (see Figure S5 and Table S1 in Supplementary Materials for more combinations of capacitance and resistance).

### 4.2. Droplet Generation

In addition to the amplitude modulation capability realized through RC constant tuning of the proposed MSS, the easy reconfigurability enabled by the Lego-like connections offers advantages for broader applications. We demonstrate here the ability of the system to remarkably improve the droplet uniformity when connected to a cross-junction microfluidic droplet generator.

**Figure 7:**
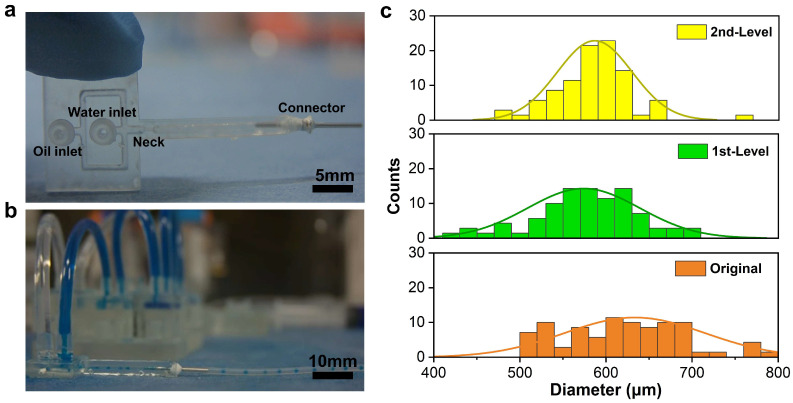
Results of droplet generation: (**a**) photograph of the cross-flow microfluidic device functioning as a water-in-oil droplet generator; (**b**) droplets generated in the cross-flow microfluidic device with the MSS shown in the background; (**c**) histograms of droplet diameter distributions for three states of the MSS.

To illustrate the impact of flowrate stabilization in microfluidic applications, we conducted droplet generation tests with original, first-level, and second-level states. Figure 7a shows a photograph of a cross-junction droplet generator. The inner square channel of the generator has a cross-section area of 1000×1000 µm. A tiny neck with a cross-section area of 500×500 µm is formed at the end of the cross-channel to facilitate droplet generation. The oil continuous phase with a viscosity of 0.65 cst (PMX-200 silicone fluid, Dow Chemical, Midland, MI, USA) and an ink-dispersed water phase were connected to the MSS before entering the droplet generator. The droplet generation in the cross-flow microfluidic device is shown in Figure 7b with the MSS in the background. Both the ink-dispersed phase and the oil-continued phase were driven at a flowrate ratio of 1:2.

The distributions of the diameter of the droplets generated with different MSS configurations were evaluated and are summarized in Figure 7c. The first level means both water and oil flows were linked to the first level MSS configuration. Similarly, the second level means both water and oil flows were linked and connected to the second-level MSS configuration. The polydispersity [26] of the droplet diameters (Table 2) was analyzed to evaluate the droplet size uniformity. In Table 2, the original state has the highest polydispersity of 0.13. As the MSS configuration changes from the original state to the second-level state, the polydispersity has improved significantly, and the best polydispersity achieved in the experiment is 0.07.

The droplet generation process is highly sensitive to flowrate fluctuations [27]. Elevated flowrates are associated with the production of smaller droplets, whereas reduced flowrates yield larger droplets [28]. The variability in flowrates engenders inconsistencies in the droplet dimensions and disrupts the regularity of droplet generation. Our system addresses these fluctuations by facilitating meticulous control and fine-tuning of hydraulic capacity and resistance. This enhanced stability culminates in a more uniform distribution of droplet sizes, as corroborated by the emergence of a well-defined normal distribution in our statistical analysis.

## 5. Conclusions

In this work, we developed a 3D-printed Lego-like pluggable MSS with controllable stabilization ratios to provide a range of discrete RC constants for fluidic circuits. A simplified three-element circuit model was derived to describe the performance of the system, and experiments were conducted to evaluate the accuracy of the circuit model. The comparison of the theoretical and experimental values of the amplitude ratio at different operating frequencies yielded an R-square correlation coefficient of 0.95, indicating that the circuit model is accurate in modeling the behavior of the MSS. For a fluid circuit, a critical parameter is the RC constant. Each RC constant of a working state of the MSS was extracted using a curve-fitting method. As a result, the fluctuation could be reduced to less than 1% with a controllable working curve. Finally, the MSS was successfully applied to stabilizing the performance of a fluidic cross-junction droplet generator device. The polydispersity of the diameter of the droplets was reduced from 0.13 to 0.07 as the MSS changed from the original state to the second-level state. The results of the droplet diameter statistics revealed that selecting an appropriate working state for the MSS can improve the uniformity of the droplet sizes. We envision that the Lego-like pluggable MSS could be further integrated into various microfluidic platforms, such as a biological cell culture microfluidic platform, to fully exploit the tunable RC constants of the MSS, where facile and flexible modulations of the flowrate amplitudes are needed.

## Figures and Tables

**Table 1:** Summary of different types of stabilizers.

Stabilizer Type	Degree of Modularity	Best Stable Flow	Best Stabilization Ratio
Single-chamber stabilizer [24]	Discrete component	50 ± 3 µL/min	6%
Multi-chamber Stabilizer [7]	Discrete components with different chamber numbers	50 ± 1.4 µL/min	2.8%
Fluidic low-pass filter [6]	Discrete components with different membranes	10 ± 0.21 mL /hour	2.1%
Hand-powered stabilizer [3]	Discrete component with modular connectors	1214 ± 22 µL/min	1.8%
Air chamber stabilizer [10]	Parallel-connected stabilizers	60 ± 5 µL/min	8%
Bubble damper stabilizer [25]	Discrete component with controllable dampers	9.64 ± 0.27 µL/min	2.8%
This work	Modular stabilizer with modular connectors	504.01 ±1.86 µL/min	0.93%

**Table 2:** Polydispersity and standard deviation of the generated droplets under different states of the MSS.

Configuration	Standard Deviation (µm)	Polydispersity
Original	81.8	0.13
First Level	62.9	0.11
Second Level	44.5	0.07

## Data Availability

The original contributions presented in the study are included in the article/Supplementary Materials, further inquiries can be directed to the corresponding author/s.

## Supplementary Materials

The following supporting information can be downloaded at https://www.mdpi.com/article/10.3390/mi15070843/s1. Figure S1: Evolution of device design from (a) to (e). (a) Luer-fitting connection, (b) screw bonding, (c) tubing-connection, (d) modular design with independent unit base, (e) modular design with Lego-like platform; Figure S2: 2D-schematic diagram: (a) the modular resistor, (b) the modular capacitor, (c) the breadboard, (d) the linker; Figure S3: An example of the transient amplitude response of the level 2 MSS system working at 0.2 Hz; Figure S4: An example of LSM fitting with R = 0.986; Figure S5: The experimental resistance of different numbers of resistors; Table S1: The experimental fluidic capacitance of different devices with different membranes (unit: $m^{3}*{Pa}^{-1}$). Ref. [8,11,29] is cited in the Supplementary materials.
