# Supplementary material for: A Lego-Like Reconfigurable Microfluidic Stabilizer System with Tunable Fluidic RC Constants and Stabilization Ratios
Source: Micromachines (Basel). 2024 Jun 28;15(7):843. doi: 10.3390/mi15070843 (PMC11278724; doi:10.3390/mi15070843)
Supplement: Supplementary file 1 [file micromachines-15-00843-s001.zip › micromachines-3070639-supplementary.pdf]

micromachines

Supplementary Information

A Lego-like reconfigurable microfluidic stabilizer system with tunable fluidic RC constants and stabilization ratios

Wuyang Zhuge [1,2,†], Weihao Li [1,3,†], Kaimin Wang [1], Zhuodan Chen [1], Chunhui Wu [1], Kyle Jiang [2,4], Jun Ding [3], Carl Anthony [2] and Xing Cheng [1*]

[1]Guangdong-Hong Kong-Macau Joint Laboratory on Micro-Nano Manufacturing Technology，Department of Material Science and Engineering, Southern University of Science and Technology, Shenzhen, Guangdong, P.R. China

[2]Department of Mechanical Engineering, University of Birmingham, Edgbaston, Birmingham, B152TT, United Kingdom

[3]Department of Materials Science and Engineering, National University of Singapore, 9 Engineering Drive 1, 117576, Singapore

[4]Yangtze Delta Region Institute of Tsinghua University，705 Yatai Road, Jiaxing, Zhejiang, China

* Correspondence: Xing Cheng (chengx@sustech.edu.cn)

† These authors contributed equally: Wuyang Zhuge, Weihao Li.

S1. Design iterations of Lego-like pluggable microfluidic system

Fig. S1 depicted the design iterations based on the feedback from experiments. With 3D printing, we could efficiently modify and test different device designs in order to figure out the optimal design for the proposed system in this work. There are three key elements when it comes to device designing: connection, bonding, and combination. The model structure using luer-fitting connection is shown in Fig. S1(a). However, this design lacks sufficient waterproofing capabilities. As demonstrated in Fig. S1(c), the luer-connector is removed and replaced by tubing connection. Such tubing connections can achieve the appropriate tightness. The screw-bonding design is shown in Fig. S1(b), six holes are utilized for screws to connect this part to a cover portion. However, because this bonding approach is unable to completely seal the entire device, photocurable resin is used to successfully bind the various parts in subsequent iterations. Figures S1(d) and S1(e) show the optimized forms of device design used in this study, which are suited for modular combinations and allow for easy reconfiguration.

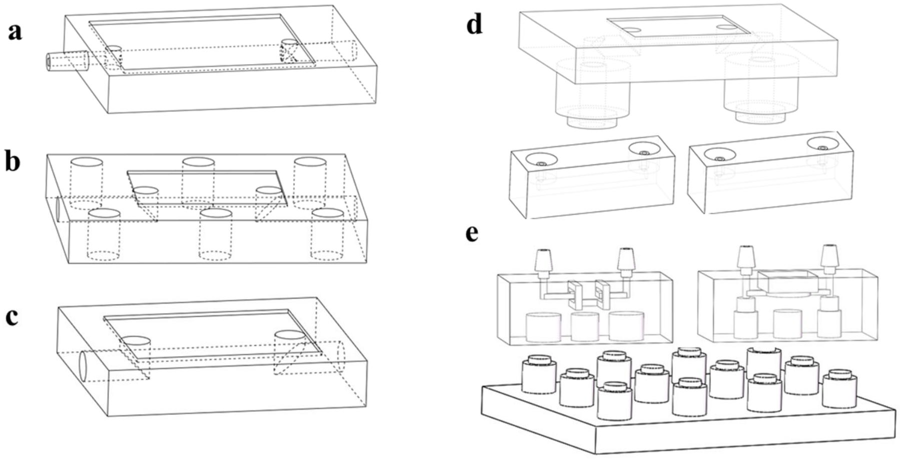

Figure S1. Evolution of device design from (a) to (e). (a) Luer-fitting connection, (b) screw bonding, (c) tubing-connection, (d) modular design with independent unit base, and (e) modular design with Lego-like platform.

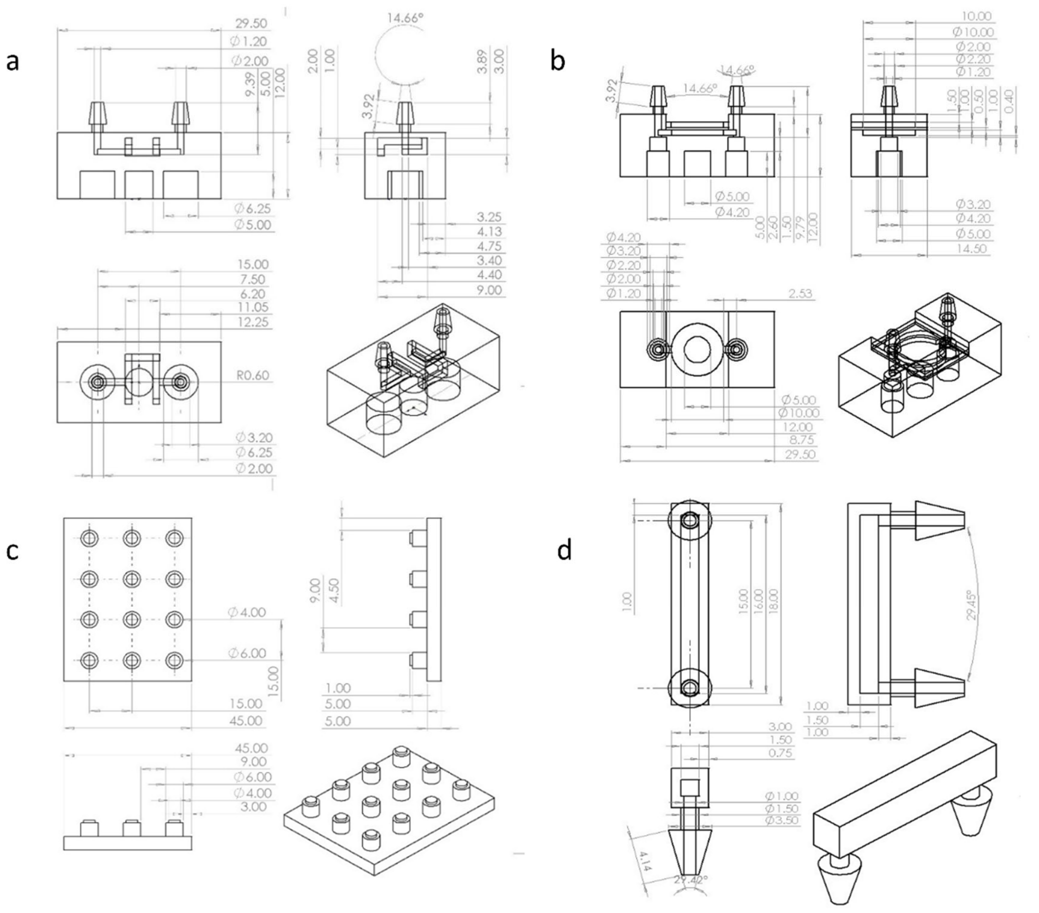

Figure S2. 2D-schematic diagram: (a) the modular resistor, (b) the modular capacitor, (c) the breadboard, (d) the linker.

S2. Simplified circuit modelling

The derivation process is shown as following. Applying Kirchhoff's current law to the two nodes (marked by blue dots) in Fig. 4(a), we get:

$$\frac{P_{in}-P_{c0}(t)}{R_0} = C_0\frac{dP_{c0}(t)}{dt} + \frac{P_{c0}(t)-P_c(t)}{R_c} \quad \text{(S-1)}$$

$$\frac{P_{c0}(t)-P_c(t)}{R_c} = C\frac{dP_c(t)}{dt} + \frac{P_c(t)}{R_{loading}} \quad \text{(S-2)}$$

Rearrange Eqn. S-2, we have:

$$P_{c0}(t) = R_cC\frac{dP_c(t)}{dt} + \frac{R_cP_c(t)}{R_{loading}} + P_c(t) \quad \text{(S-3)}$$

Applying the Kirchhoff's current law to the node (marked by a blue dot) in the simplified circuit model described in Fig. 4(b) yields the following equation:

$$\frac{P_{in}-P_c(t)}{R_1} = C_{overall}\frac{dP_c(t)}{dt} + \frac{P_c(t)}{R_2} \quad \text{(S-4)}$$

In order to justify the correctness of the simplification, we could plug Eqn. S-3 into Eqn. S-1, we have:

$$\frac{P_{in}-R_cC\frac{dP_c(t)}{dt}-\frac{R_cP_c(t)}{R_{loading}}-P_c(t)}{R_0} = C_0\frac{d\left(R_cC\frac{dP_c(t)}{dt}+\frac{R_cP_c(t)}{R_{loading}}+P_c(t)\right)}{dt} + C\frac{dP_c(t)}{dt} + \frac{P_c(t)}{R_{loading}} \quad \text{(S-5)}$$

Reorganize Eqn. S-5, we have:

$$\frac{P_{in}-P_c(t)}{R_0} = \left(\frac{R_c}{R_0}C + C_0\frac{R_c}{R_{loading}} + C_0 + C\right)\frac{dP_c(t)}{dt} + P_c(t)\left(\frac{1}{R_{loading}} + \frac{R_c}{R_{loading}R_0}\right) + R_cCC_0\frac{d^2P_c(t)}{dt^2} \quad \text{(S-6)}$$

Neglecting the last second-order differential terms, Eqn. S-6 has the same form as Eqn. S-4 with $C_{overall} = \left(\frac{R_C}{R_0}C + C_0\frac{R_C}{R_{loading}} + C_0 + C\right)$.

The hydraulic resistance/capacitance equation is given by[1-3]:

$$R = \frac{\Delta P_f}{Q} = \frac{8\eta L}{\pi r^4} \quad \text{(S-7)}$$

$$C_0 = \frac{dV_f}{dP_f} = \frac{3r^6(1-P_r{}^2)\pi}{64h^3E} \quad \text{(S-8)}$$

where:

R is the fluidic resistance,
Q is the flow rate,
C_0 is the fluidic capacitance,
V_f is the fluid volume,
P_f is the pressure drop,
r is the radius of the membrane,
h is the thickness of the membrane.
E is the Youngs modulus,
P_r is the Poisson's ratio.

These equations describe the relationship between the pressure drop ΔP and the flow rate Q in a cylindrical pipe with length L, radius r and liquid viscosity η, which is relevant to the hydraulic channels in our system. For example, apply the Hagen-Poiseuille equation into equation 1, $P_{in} - P_c(t)$ is the pressure drop ΔP, thus $\frac{P_{in}-P_c(t)}{R_1}$ means the flow rate Q of the fluidic circuit. The resistance can be extracted by using the experimental pressure difference ΔP divided by flow rate Q. The hydraulic capacitance can be calculated by using extracted RC constant divided by experimental resistance.

S3. Extraction of RC constants

$$Q(T) = (Q_{avg} - Q_L)\exp\left(-\frac{T-T_0}{\tau}\right) \quad \text{(S-9)}$$

Q(T) is the flowrate of the system at time T, Q_{avg} is defined as the average flowrate of the working state of the MSS, Q_L refers to the low point of the flowrate when the pump is stopped, which also represents the flowrate driving by the pressure drop due to gravity (height difference between the inlet and outlet of the experimental setup). T_0 is the cutoff time of the input signal. The transient behavior of the fluidic system is characterized by a time constant τ that equals to the value of resistor R times the value of capacitor C, which is the response time of the fluidic system. In Fig. S3, the data illustrates the ramp-down response of the MSS. With an initial pumping pressure oscillating at 0.2Hz, the average flowrate of the microfluidic channel is locked at Q_{avg}=520 ± 0.171 µl/min. The input signal cuts off at T_0, and finally the flowrate of the system drops to Q_L=0. By reforming Eqn. (S-9), we can get Eqn. (S-10) as follow:

$$ln\left(\frac{Q(T)}{Q_{avg}-Q_L}\right) = -\frac{(T-T_0)}{\tau} \quad \text{(S-10)}$$

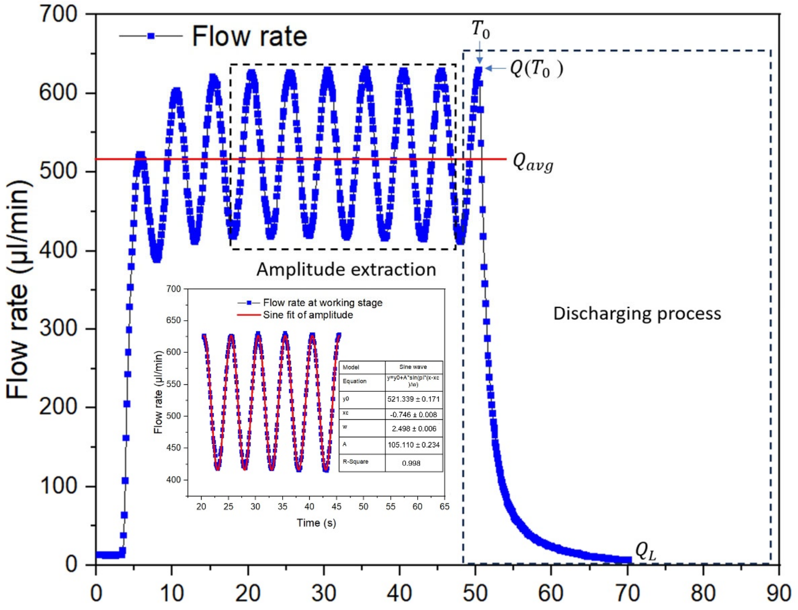

Figure S3. An example of the transient amplitude response of the level 2 MSS system working at 0.2Hz.

The amplitude response of level 2 stabilizer is extracted during the working stage of the system. The fitted amplitude is 105.108 ± 0.239 μl/min, thus the stabilization ratio at this point is calculated as 0.525. The RC constant can be extracted during the discharging process by using equation. S-10. The result is shown in Figure. S3 and Figure. S4.

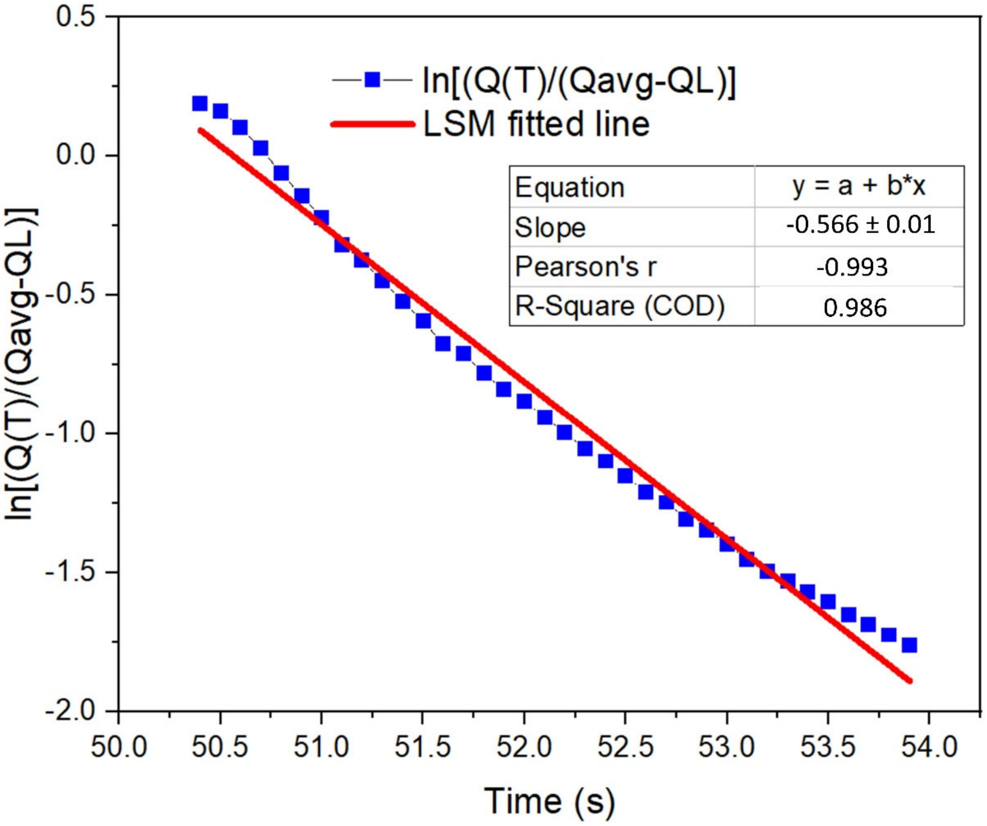

Figure S4. An example of LSM fitting with R = 0.986.

In Figure. S4, an example of least-square method (LSM) fitting according to Eqn. S-10 is given. The MSS contains 2 levels of stabilizer. The slope of the curve refers to $-\frac{1}{\tau} = -0.5667$. The RC constant τ is calculated as 1.76s when working frequency is 0.2 Hz. The mean value of the extracted RC constant is used to calculated the theoretical working curve of the MSS.

To provide customized combinations of stabilizers, we also tested the resistance and capacitance of the device by changing the number of devices, the thickness of the membrane and the diameter of the chamber to provide different capacitance and resistance.

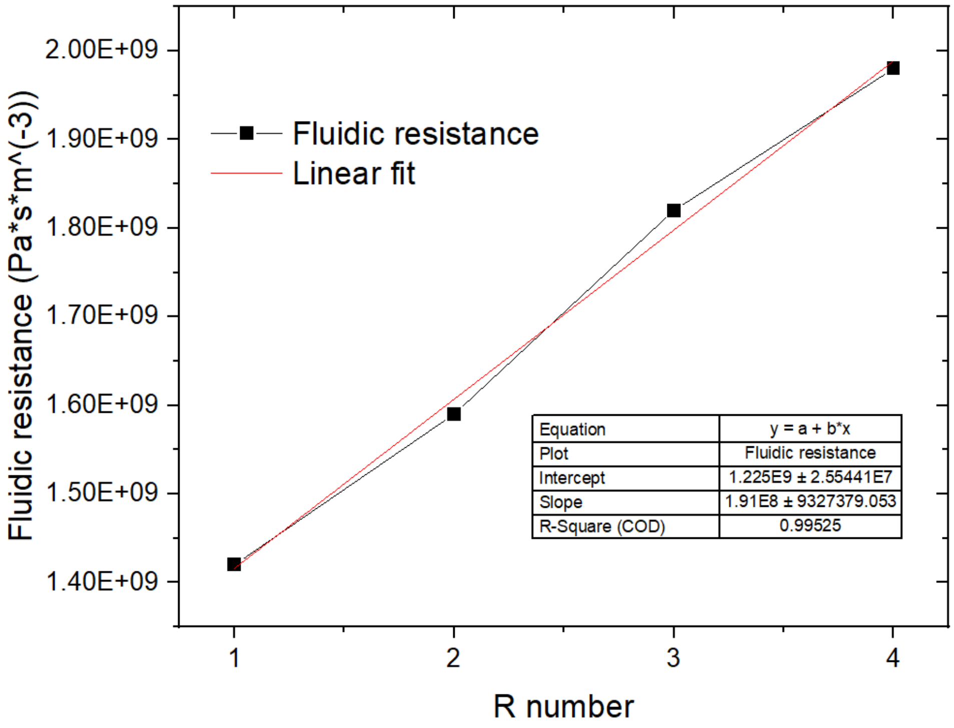

Figure S5. The experimental resistance of different numbers of resistors.

Fig. S5 provides the experimental resistance of different numbers of resistor, the results show a linear relationship with the number of resistors. From the slop, it can be calculated that the device provides an average resistance of $1.91 \times 10^8 Pa * s * m^{-3}$. By adding different numbers of resistors, the resistance of the system can be controlled.

Table S1. The experimental fluidic capacitance of different devices with different membranes (unit: $m^3 * Pa^{-1}$).

Diameter (row) / Thickness (column)	6mm	8mm	10mm
300μm	$2.78^{-10} \pm 5.05^{-12}$	$7.78^{-10} \pm 6.57^{-11}$	$1.35^{-9} \pm 8.08^{-11}$
800μm	$1.82^{-10} \pm 6.57^{-11}$	$4.09^{-10} \pm 7.58^{-11}$	$7.53^{-10} \pm 3.03^{-11}$
1000μm	$1.01^{-10} \pm 4.04^{-12}$	$2.37^{-10} \pm 2.02^{-11}$	$3.43^{-10} \pm 1.01^{-11}$

Table. S1 provides multiple fluidic capacitors by changing the diameter and thickness of the membrane to give different capacitance. The level 2 stabilizer in the manuscript provided an optimal balance between stabilization time and stability. If other RC constant is required, user can link different types of capacitors or different numbers of resistors to

provide more or less levels of stabilizing combinations with different stabilization ratios and RC constants.

Reference

1. Iyer V, Raj A, Annabattula RK, Sen AK. Experimental and numerical studies of a microfluidic device with compliant chambers for flow stabilization. JOURNAL OF MICROMECHANICS AND MICROENGINEERING. 2015;25(7).
2. Wu C-H, Chen C-W, Kuo L-S, Chen P-H. A Novel Approach to Measure the Hydraulic Capacitance of a Microfluidic Membrane Pump. Advances in Materials Science and Engineering. 2014;2014:1-8.
3. Lee J, Rahman F, Laoui T, Karnik R. Bubble-induced damping in displacement-driven microfluidic flows. PHYSICAL REVIEW E. 2012;86(2).
